# Temporary pacemaker implantation via median cubital vein: A simple safe and effective technique

**DOI:** 10.1002/clc.24097

**Published:** 2023-07-31

**Authors:** Dehua He, Ziguan Zhang, Huiqing Huang, Kaimin Lin, Yan Ge, Xiongbiao Lin, Qiang Xie, Weihua Li, Zhengrong Huang

**Affiliations:** ^1^ Department of Cardiology, Xiamen Key Laboratory of Cardiac Electrophysiology, Xiamen Institute of Cardiovascular Diseases, The First Affiliated Hospital of Xiamen University, School of Medicine Xiamen University Xiamen Fujian China; ^2^ First Department of Geriatric Medicine The First Affiliated Hospital of Xiamen University Xiamen Fujian China; ^3^ Department of Echocardiography The First Affiliated Hospital of Xiamen University Xiamen Fujian China; ^4^ Department of Electro‐Cardiographic Information The First Affiliated Hospital of Xiamen University Xiamen Fujian China

**Keywords:** arrhythmia, median cubital vein, temporary pacemaker implantation

## Abstract

**Background:**

Temporary cardiac pacemaker implantation (PM) via the femoral and subclavian veins is widely used in clinics to treat patients with severe bradycardia or tachycardia, but it is technically challenging and potentially associated with various complications.

**Hypothesis:**

This study investigated the feasibility and safety of a novel method of PM implantation via the median cubital vein.

**Methods:**

A total of 279 patients of the First Affiliated Hospital of Xiamen University between March 2020 and December 2021 who required no‐emergency PM implantation were enrolled. The patients were divided into three groups based on the temporary PM implantation routes: F‐control (*n* = 107), via the femoral vein; S‐control (*n* = 67), via the subclavian vein, and N‐group (*n* = 105), via the median cubital vein. The sheath placement time (SPT), electrode placement time (EPT), electrode arrival rate (EAR), rate of sensing and pacing (RSP), radiation quantity (RD), electrode dislocation rate (EDR) and average electrode retention time (AERT) were recorded and evaluated. In addition, the Hamilton Anxiety Scale (HAMA) and Self‐Rating Depression Scale (SDS) were used to evaluate the comfort levels of patients in the three groups.

**Results:**

There were no significant differences between the groups with regard to age, EAR, RSP, EPT, RD, and AERT (*p* > 0.05). However, the N‐group had significantly lower SPT than the F‐control and S‐control groups (67.0 ± 22.0 s vs. 321.7 ± 122.2 s and 307.3 ± 128.5 s, *p* = 0.000). Additionally, the F‐control had significantly higher EDR than the S‐control group and the N‐group (11 (10.3%) vs. 2 (3.0%) and 3 (2.9%), *p* = 0.036). Besides, comparison of the HAMA and SDS scores before and after PM implantation showed significant differences in the S‐control group (*p* = 0.010) and the N‐group (*p* = 0.000).

**Conclusions:**

Temporary PM implantation via the median cubital vein is safe, effective, and less time‐consuming.

## Graphical abstract

According to the Inclusion criteria and Exclusion criteria, a total of 279 patients who required no‐emergency temporary pacemaker (PM) implantation were included in this study and were assigned to different groups according to the route of PM implantation.

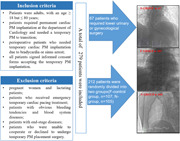

## 1 INTRODUCTION

Cardiac arrhythmias are some of the most common cardiac diseases and the prevalence has been increasing with the expansion of the aging population.^1^, ^2^ Cardiac arrhythmias may be managed with different interventions, depending on the type of arrhythmia.^3^ One of the medical interventions available is pacemaker (PM) implantation. Cardiac PM implantation was first performed in a patient in 1958, though the implant only lasted for a few hours.^4^ However, with more understanding of the molecular basis underlying cardiac arrhythmias and advancements in implantation technology, permanent cardiac PM implantation has become the mainstay treatment for severe arrhythmias such as atrial fibrillation and atrioventricular block,^5^ which are two common cardiac arrhythmias that often coexist with sinus node dysfunction.^6^ In addition to permanent cardiac PM, temporary cardiac PM implantation is also commonly used in clinics to treat patients with severe bradycardia or tachycardia, with the aim of restoring compromised hemodynamics until systemic symptoms are resolved and/or permanent cardiac PM implantation can be performed.^7^

To perform temporary cardiac PM implantation, venous access routes are important because they are associated with the rates and severity of potential complications. Two commonly used venous access routes are the femoral and subclavian veins.^8^ It is widely recognized that temporary cardiac PM implantation through these two veins benefits patients who experience rhythm disturbances, conduction delays, or low cardiac output due to bradycardia.^9^ However, the femoral and subclavian veins are both deep veins; therefore, puncturing through them is technically challenging and potentially associated complications can easily occur.^10^ For example, pneumothorax, which develops when air enters the space between the lungs and the chest wall as a result of injury and may lead to partial or full loss of lung function, is one of the complications linked to subclavian venipuncture.^10^ On the other hand, femoral vein puncture can easily slip into the femoral artery, causing hematoma or arteriovenous fistula.^10^ Hence, there is a need to identify other appropriate venous access site(s) that may be easier to use with fewer and/or milder complications.

Here, we report a novel method for the implantation of temporary PMs via the median cubital vein, a superficial vein. Compared with the other two venous access routes, puncturing through the median cubital vein is quicker, simpler, and more effective, with fewer complications. Our findings suggest that this new venous access route should be widely used for temporary PM implantation in clinical practice.

## 2 PATIENTS AND METHODS

### 2.1 Patient selection

**Figure 1:**
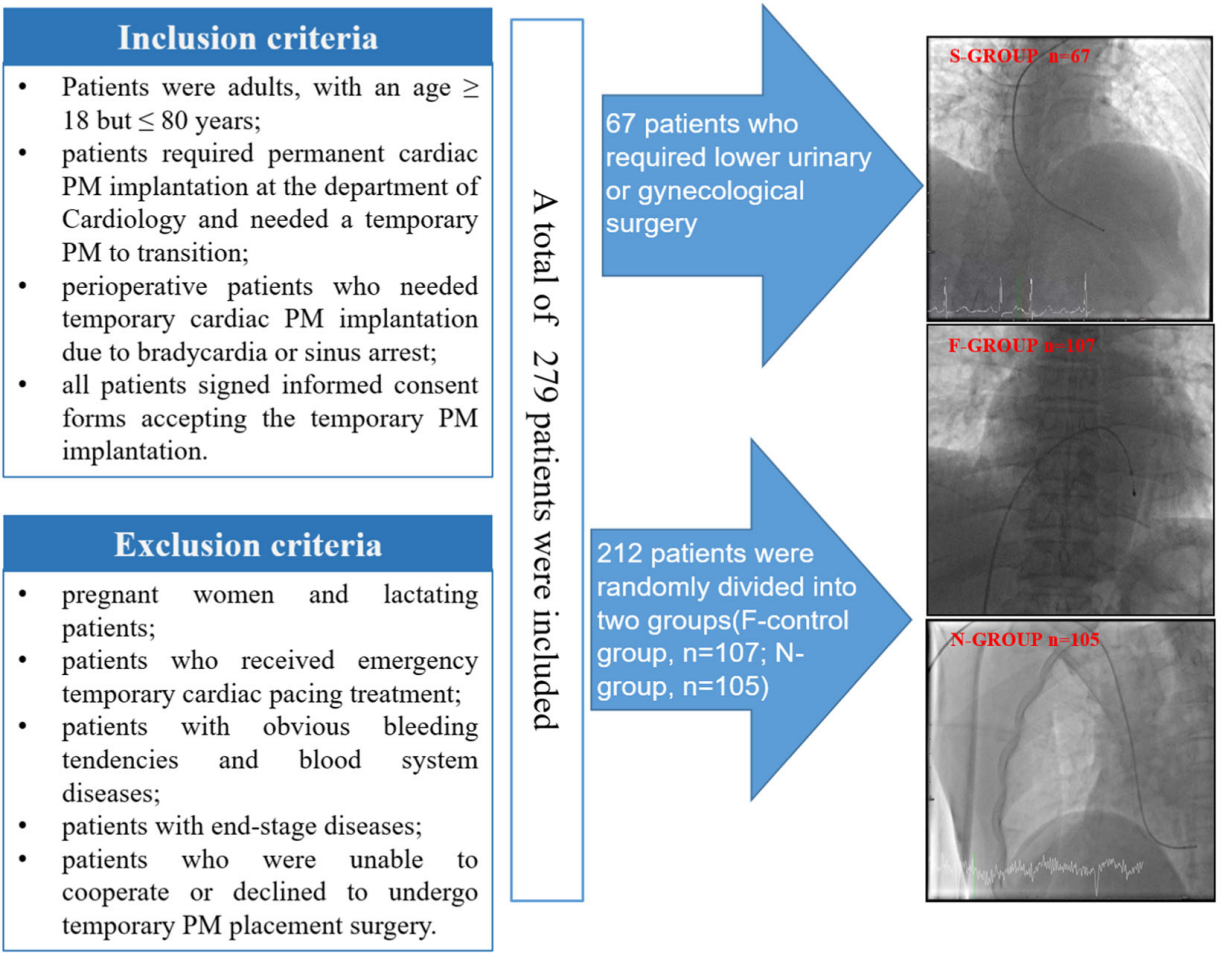
Flowchart of patients selection and assignment. According to the Inclusion criteria and Exclusion criteria, a total of 279 patients who required no‐emergency temporary PM implantation were included in this study and were assigned to different groups according to the route of PM implantation. PM, pacemaker.

A total of 279 patients who required no‐emergency temporary PM implantation at the Inpatient Department of the First Affiliated Hospital of Xiamen University between March 2020 and December 2021 were included in this study. These patients were assigned to different groups according to the route of PM implantation: 67 patients who required lower urinary or gynecological surgery were assigned to the subclavian vein route as S‐control group, while those with no special path limits were randomly divided into two groups for the femoral vein route (F‐control group, *n* = 107) and the median cubital vein route (N‐group, *n* = 105) (Figure 1).

All patients underwent temporary PM implantation in the catheterization room. The conventional method was used and electrodes were placed under X‐ray fluoroscopy for the S‐ and F‐control groups.^10^ Indwelling needles were embedded in the median cubital vein in the ward by specialist nurses. The subsequent surgical procedures were performed by doctors in the catheterization room and electrodes were placed under X‐ray fluoroscopy. The sheath placement time (SPT), electrode placement time (EPT), electrode arrival rate (EAR), rate of sensing and pacing (RSP), radiation dose (RD), and complications were recorded. The follow‐up time was 1 week after the temporary PM was implanted.

**Inclusion criteria** ① Patients were adults, with an age ≥18 but ≤80 years; ② Patients required permanent cardiac PM implantation at the department of Cardiology and needed a temporary PM to transition; ③ Perioperative patients who needed temporary cardiac PM implantation due to bradycardia or sinus arrest; ④ All patients signed informed consent forms accepting the temporary PM implantation.

This study was approved by the Ethics Committee of the First Affiliated Hospital of Xiamen University (Approval Number: 2020038). All patients provided written informed consent for their anonymized medical data to be analyzed and published for research purposes.

**Exclusion criteria** ① Pregnant women and lactating patients; ② Patients who received emergency temporary cardiac pacing treatment; ③ Patients with obvious bleeding tendencies and blood system diseases; ④ Patients with end‐stage diseases; ⑤ Patients who were unable to cooperate or declined to undergo temporary PM placement surgery.

### 2.2 Surgical procedure for temporary PM implantation in control groups

The operating area was disinfected and locally anesthetized with 1 mL 1% lidocaine. A guide wire was inserted after puncturing the femoral or subclavicular vein (Supporting Information: Figure S1A–C). Afterward, the puncture needle was removed, and a 6 F sheath tube was inserted along the guide wire (Terumo Company) (Supporting Information: Figure S1D–E). Next, a temporary PM electrode was delivered to the right ventricular apex through the vein system (Supporting Information: Figure S1F). Finally, X‐ray fluoroscopy was performed to fix the electrode head position, an external PM was connected, the sheath tube was removed, and the local suture was fixed (Supporting Information: Figure S1G–J for the S‐control group and Supporting Information: Figure S1H,K,L for the F‐control group).

### 2.3 Surgical procedure for temporary cardiac PM implantation via median cubital vein

**Figure 2:**
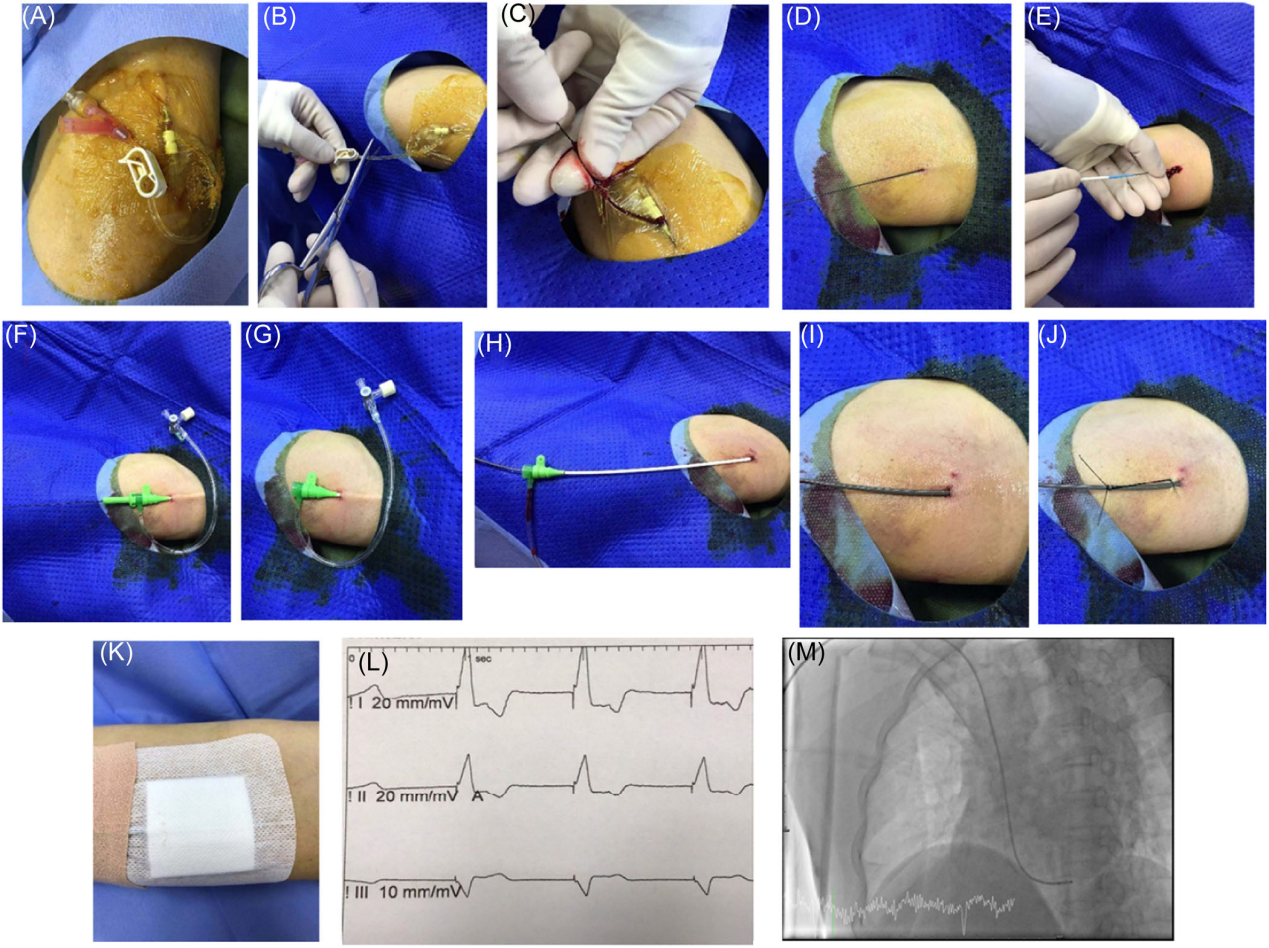
Temporary PM implantation procedure via median cubital vein. (A) Embedded indwelling needle in the median cubital vein. (B) Cut off the soft outer sleeve. (C) Inserted the guide wire along the outer sleeve into the vein. (D) Withdrawn the indwelling needle. (E–G) A sheath was inserted into the blood vessel. (H–K) A temporary PM electrode was inserted through the sheath tube and fixed. (L) An electrocardiogram (ECG) for the PM. (M) X‐ray fluoroscopy was performed to fix the electrode head position. PM, pacemaker.

(1) Embedding indwelling needle in the median cubital vein
  An indwelling needle was routinely embedded in the median elbow vein in the ward by specialist nurses (specification: 1.1 × 30 mm, 20 G; Shanghai Kent Lai group) (Figure 2A).
(2) Disinfection of the operating area, placement of sterile towels, and local anesthesia administration in the Cardiac Catheterization Room.

The indwelling needle consisted of a stainless steel core, a soft outer sleeve, and a plastic base. After disinfection and placing sterile drapes, local anesthesia was administered with 1% 1 mL lidocaine for the surrounding skin. The soft outer sleeve was then cut off, leaving about 2 cm (Figure 2B).

③ PM electrode placement

The guide wire was inserted along the outer sleeve into the vein for about 10 cm (Figure 2C and Supporting Information: Videos) and the indwelling needle was withdrawn. During the withdrawal process, the guide wire was retained in the original position of the blood vessel (Figure 2D). A sheath (6 F; Terumo Company) was inserted into the blood vessel along the guide wire until the entire sheath was in the vessel, and the guide wire was then removed (Figure 2E,F). Next, the temporary PM electrode was inserted through the sheath tube, basilic veins, subclavian vein, superior vena cava, and right atrium to the appropriate position in the right ventricular apex, which was confirmed when the electrocardiogram (ECG) showed the PM nail and pacing with a wide QRS wave. Finally, the sheath tube was carefully withdrawn, and the electrode and temporary PM were fixed (Figure 2H–M).

### 2.4 Anxiety and depression evaluation

We conducted questionnaire surveys for the enrolled patients using the Hamilton Anxiety Rating Scale (HAMA) and Self‐Rating Depression Scale (SDS) to evaluate anxiety and depression. A HAMA score over 29 indicates high anxiety, over 21 indicates significant anxiety, over 14 indicates moderate anxiety, over seven indicates low anxiety, and below seven indicates no anxiety. For SDS, the 20 items were summed to obtain a total score and the formula conversion *Y* = in + (1.25×) was used to obtain the standard total score (*Y*). The cut‐off value for depression with SDS was 41 points for the total score and 53 points for the standard score.

### 2.5 Definition

#### 2.5.1 SPT

The time from the administration of local anesthesia to successful placement of the sheath tube.

#### 2.5.2 EPT

The time from when the electrode entered the sheath tube in the median cubital vein to the time when the operating electrode was in the right position and the ECG indicated that the temporary PM pace was well‐driven.

#### 2.5.3 EAR

Accurate positioning for the electrode was defined as the fixation of the right ventricular apex by the electrode tip under X‐ray fluoroscopy, confirmed when the ECG indicated that the temporary PM pace was well‐driven. EAR (%) = the number of cases with the electrode in the right position/the total number of cases in the group.

#### 2.5.4 Sensing and pacing

The temporary PM sensed the frequency and drive according to a set frequency. A typical ECG is shown in Figure 2L.

#### 2.5.5 RQ

The frame frequency was set at 7.5 p/s. The fluoroscopy mode was used during the surgical procedure and images obtained under this mode were saved. For each patient, images of the anteroposterior and left anterior oblique 30° were retained to confirm the PM electrode position. The digital subtraction angiography system automatically calculated the total amount of radiation for each patient upon completion of the operation.

#### 2.5.6 Electrode dislocation

During the period of temporary PM implantation, the occurrence of transient, intermittent, continuous PM perception, or poor drive was defined as electrode dislocation, excluding factors such as battery depletion and the PM itself.

### 2.6 Statistical analysis

Continuous variables with normal distribution were expressed as mean ± standard deviation (SD) and analyzed with the Student *t*‐test. Categorical variables were expressed as percentage and analyzed using the *χ* ^2^ test. All analyses were conducted using SPSS version 17.0 (SPSS Inc.). Statistical significance was set at *p* < 0.05.

## 3 RESULTS

### 3.1 Comparison of demographic and basal clinical characteristics of patients between the three groups

A total of 279 patients were included in this study and were divided into three groups: F‐control (*n* = 107), S‐control (*n* = 67), and N‐group (*n* = 105). None of these patients required emergency PM implantation. The details for the basic characteristics of all participants in these three groups are listed in Supporting Information: Table S1. There were no significant differences in demographic and basal clinical characteristics including age, gender, renal function (creatinine, blood urea nitrogen [BUN]), myocardial enzymonram (myoglobin, creatine kinase [CK]‐MB, lactate dehydrogenase [LDH]), hematology (white blood cells [WBC], hemoglobin [HBG], platelets [PLT]), cardiac function index (brain natriuretic peptide [BNP]), and the incidence of sinus bradycardia and II°II atrioventricular block (AVB) between the three groups (all *p* > .5), indicating that the three methods evaluated were all safe.

### 3.2 Comparison of laboratory results between the three groups after temporary PM implantation

After temporary PM implantation, the laboratory tests were repeated to evaluate the safety of the methods. As shown in Supporting Information: Table S2, there were no significant differences between the groups with regard to the renal function (creatinine, BUN), myocardial zymogram (myoglobin, CK‐MB, LDH), hematology (WBC, HBG, PLT), and cardiac function index (BNP).

### 3.3 Comparison of operation and safety parameters between the three groups

The operation parameters in the three groups are outlined in Table 1. There were no significant differences in EPT, EAR, RSP, and RD between the groups. However, the N‐group had a significantly lower SPT than the F‐control and S‐control groups (67.0 ± 22.0 s vs 321.7 ± 122.2 s and 307.3 ± 128.5 s, *p* = 0.000).

**Table 1:** Comparison of operation and safety parameters between groups.

	F‐control (*n* = 107)	S‐control (*n* = 67)	N‐group (*n* = 105)	*p* Value
EPT (Sec)	79.1 ± 19.6	78.3 ± 19.9	78.7 ± 20.4	0.958
EAR (%)	107 (100)	67 (100)	105 (100)	
RSP (%)	107 (100)	67 (100)	105 (100)	
RD (mGy)	18.7.7 ± 9.3	19.4 ± 8.2	17.9 ± 6.8	0.471
SPT (Sec)	321.7 ± 122.2	307.3 ± 128.5	67.0 ± 22.0	0.000
AERT (day)	3.0 ± 1.5	2.9 ± 1.4	3.4 ± 1.5	0.123
EDR (%)	11 (10.3)	2 (3.0)	3 (2.9)	0.036
local ecchymosis	6 (5.6)	1 (1.5)	2 (1.9)	0.204
artery puncture	13	3	0	

*Note*: Data are *n* (%) or mean ± SD. Data analysis was performed using the independent samples *t*‐test.

Abbreviations: AERT, Average electrode retention time; EAR, electrode arrival rate; EDR, Electrode dislocation rate; EPT, electrode placement time; RD, radiation dose; RSP, rate of sensing and pacing; SPT, sheath placement time.

The details of the safety analysis of three groups of patients are shown in Table 1. The average electrode retention time (AERT) for the F‐control group, S‐control group, and N‐group were 3.0 ± 1.5, 2.9 ± 1.4, and 3.4 ± 1.5 days, respectively (*p* = 0.123). However, the F‐control group had a significantly higher electrode dislocation rate (EDR) than the N‐group and the S‐control group (11 (10.3%) vs. 2 (3.0%), and 3 (2.9), *p* = 0.036). Hence, the N‐group and S‐control group were safer than the F‐control group with regard to the dislocation rate.

### 3.4 Comparison of HAMA and SDS scores among the three groups

As shown in Table 2, the HAMA scores for the F‐control group, S‐control group, and N‐group were 24.6 ± 10.5, 24.4 ± 11.6, and 24.0 ± 11.3 before PM implantation (*p* = 0.913) and 22.0 ± 11.0, 19.2 ± 8.2, and 17.0 ± 9.5 after implantation (*p* = 0.002), respectively. The corresponding SDS scores were 48.3 ± 19.5, 48.1 ± 22.1, and 48.5 ± 19.4 before PM implantation (*p* = 0.990) and 42.8 ± 21.2, 38.4 ± 21.4, and 36.6 ± 20.5 after implantation (*p* = 0.086), respectively. The values before and after PM implantation were significantly different in the S‐control group (*p* = 0.010) and the N‐group (*p* = 0.000).

**Table 2:** Comparison of HAMA scores and SDS scores between groups.

	F‐control (*n* = 107)	S‐control (*n* = 67)	N‐group (*n* = 105)	*p* Value
HAMA				
Before	24.6 ± 10.5	24.4 ± 11.6	24.0 ± 11.3	0.913
After	22.0 ± 11.0	19.2 ± 8.2	17.0 ± 9.5	0.002
*p* Value	0.072	0.003	0.000	
SDS				
Before	48.3 ± 19.5	48.1 ± 22.1	48.5 ± 19.4	0.990
After	42.8 ± 21.2	38.4 ± 21.4	36.6 ± 20.5	0.086
*p* Value	0.051	0.010	0.000	

*Note*: Data are mean ± SD. Data analysis was performed using the independent samples *t*‐test.

Abbreviations: HAMA, Hamilton Anxiety Rating Scale; SDS, Self‐Rating Depression Scale.

**Figure 3:**
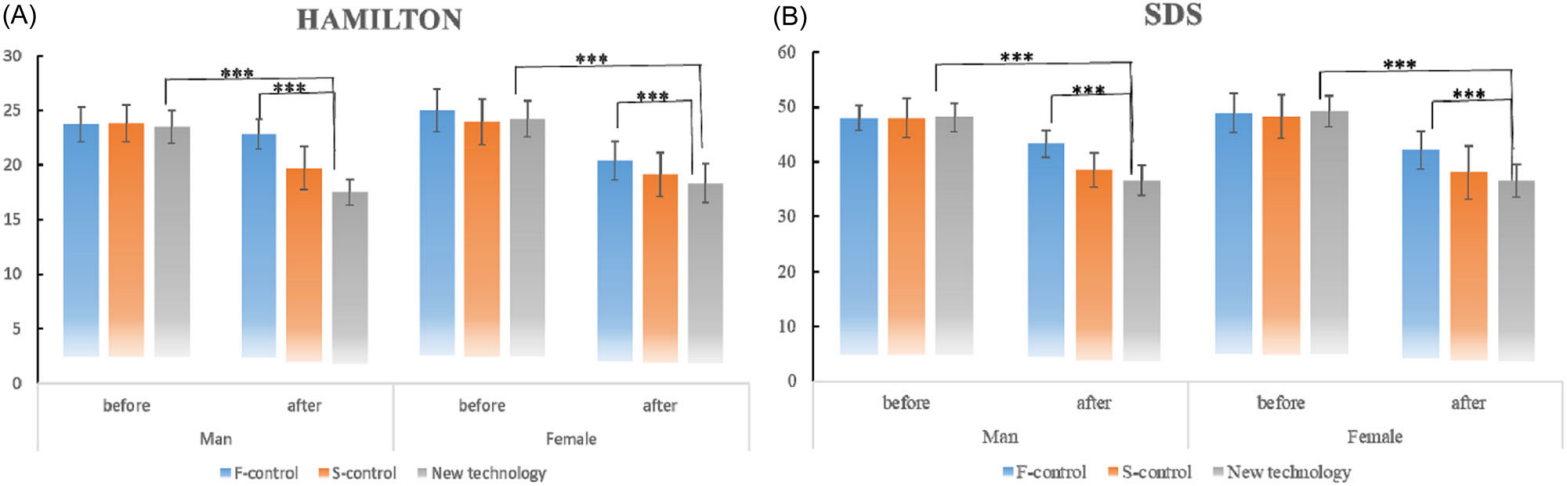
Comparison of Hamilton Anxiety Rating Scale and Self‐Rating Depression Scale scores among the three groups. (A) Comparison of Hamilton Anxiety Rating Scale scores among the three groups. (B) Comparison of Self‐Rating Depression Scale scores among the three groups.

These two psychological scale scores were further analyzed by gender (as shown in Figure 3A,B). There were no significant differences in the preoperation and postoperation HAMA and SDS scores with regard to gender. The N‐group had significantly lower HAMA and SDS scores after surgery than the F‐control group, especially among women.

## 4 DISCUSSION

Temporary cardiac PM implantation is widely used, not only for the emergency pacing of serious bradycardia, cardiac arrest or III° AVB, but also for the perioperative protection of patients with sick sinus syndrome.^7^ Traditionally, temporary PM implantation is performed through deep veins, including the femoral and subclavian veins, but the right internal jugular vein has been used in a few cases.^8^ Due to the application of bedside ultrasound and the maturity of deep‐vein puncture technology, deep‐vein puncture is safer and more effective than ever. However, challenges still exist with deep‐vein puncture and complications still occur in some patients. In the present study, we proposed, for the first time, a venipuncture approach via the middle cubital vein, a superficial vein, for the implantation of a temporary PM.

In this study, we compared the safety and efficacy of three routes for vein puncture: the femoral vein (F‐control group), the subclavian vein (S‐control group), and the median cubital vein (N‐group). We found that there were no significant differences between these groups in laboratory test results before and after PM implantation, indicating that PM implantation through these routes was safe and did not negatively affect kidney function, cardiomyocyte viability, cardiac function, and hematology.

We used a number of operational parameters, including EPT, EAR, RSP, and RD, to evaluate the effectiveness of the three routes. While we did not observe any significant differences in EPT, EAR, RSP, and RD between the groups, we observed that SPT, an indicator that is frequently used to assess the effectiveness of the PM implantation procedure,^10^ was the shortest in the N‐group, suggesting that puncture through the median cubital vein is more effective. However, the N‐group and S‐control group had significantly lower EDRs than the F‐control group. Possible reasons for this observation were that it was difficult to keep the leg in the appropriate position in the F‐control group and it was easy to dislocate the electrode due to involuntary leg bending or movement by the patient, which further highlighted the convenience of our new method.

We further analyzed the safety of the three methods. In the F‐control group, femoral artery puncture occurred in 13 patients during the procedure and local ecchymosis occurred in six patients. In the S‐control group, subclavian artery puncture occurred in three patients and local ecchymosis occurred in one patient. However, in the N‐group, local petechiae occurred in two patients. And no hematoma, pneumothorax, pseudoaneurysm, arteriovenous fistula, or thrombosis happened in our study. Taken together, these observations indicated that the novel vein puncture method had fewer and milder side effects than the conventional approaches and was a safe option.

The deep veins used for puncture in the F‐control and S‐control groups are potentially associated with a variety of complications, including artery puncture, hemopneumothorax, infection, hemorrhage, hematoma, pseudoaneurysm, arteriovenous fistula, thrombosis, vasculitis, nerve injury, and ecchymosis.^11^ Among these complications, hemopneumothorax, pseudoaneurysm, arteriovenous fistula, and nerve injury are serious, and once they occur, patients require immediate therapeutic interventions.^12^ However, the novel puncture route reported in this study was through the median cubital vein, which is a superficial vein, and the complications associated with the procedure such as subcutaneous hematoma, thrombus, ecchymosis, infection, and hemorrhage, are rare and mild. Therefore, compared to the other two routes, puncturing through the median cubital vein is safer.

In the present study, we analyzed two psychological scales, HAMA and SDS, for the patients in these three groups. HAMA is widely used to assess the responses of patients, mainly anxiety, to medical interventions,^13^ and has been demonstrated to be valid and reliable in clinical practice,^14^, ^15^ while SDS is a self‐report questionnaire widely used as a screening tool to determine symptoms linked to depression. SDS was first proposed in 1965,^16^ and was thereafter validated in a number of studies demonstrating its reliability and practicability.^17^, ^18^ The HAMA and SDS scores for both the S‐control group and the N‐group after PM implantation were much lower than the pre‐PM implantation values, suggesting that patients in these groups had less anxiety and depression than those in the F‐control group. Moreover, after PM implantation, the N‐group had the lowest HAMA score, indicating that patients in the N‐group had the least anxiety. Hence, the puncture route through the subclavian vein causes much less anxiety and depression to patients compared to the other two routes and therefore was more comfortable and acceptable for patients.

The limitations of our study should be noted. For example, our study was performed in a single center with a limited number of cases. Therefore, the findings from this study should be further corroborated in multicenter large‐cohort studies in the future.

## 5 CONCLUSION

In conclusion, although the current technology for temporary PM implantation is mature, deep‐vein puncture still has a number of disadvantages, including the need for skilled operators and potential complications. Temporary cardiac PM implantation via the median cubital vein has several significant advantages over the conventional subclavian and femoral vein approaches. First, the puncture is easier because the median cubital vein is relatively superficial. Second, puncture is safer because the procedure is associated with much fewer and milder complications. Third, puncture is more efficient because it is easier and the period from the puncture time to the EPT is greatly shortened, resulting in more time for rescue if necessary. Hence, we concluded that puncture through the median cubital vein for temporary cardiac PM implantation is simpler, safer, and more effective and should be popularized.

## AUTHOR CONTRIBUTIONS

**Dehua He, Ziguan Zhang**: Conceptualization; data curation; formal analysis; investigation; methodology; validation; writing—review and editing. **Huiqing Huang**: Conceptualization; data curation; formal analysis; resources; software; visualization; writing—review and editing. **Kaimin Lin, Yan Ge, Xiongbiao Lin, Qiang Xie**: Conceptualization; data curation; formal analysis; writing—original draft; writing—review and editing. **Weihua Li, Zhengrong Huang**: Conceptualization; data curation; formal analysis; funding acquisition; project administration; supervision; writing—original draft; writing—review and editing.

## CONFLICT OF INTEREST STATEMENT

The authors declare no conflict of interest.

## Supporting information

Supporting information.

Supporting information.

Supporting information.

## Data Availability

The data of this study are available from the corresponding author upon reasonable request.
