# Supplementary material for: Temporary pacemaker implantation via median cubital vein: A simple safe and effective technique
Source: Clin Cardiol. 2023 Jul 31;46(10):1268–75. doi: 10.1002/clc.24097 (PMC10577567; doi:10.1002/clc.24097)
Supplement: Supplementary file 3 — Supporting information. [file CLC-46-1268-s001.docx]

**Supplementary figure 1. Temporary PM implantation procedure via femoral vein (A-J) and subclavian vein (A-H, KL).**

**
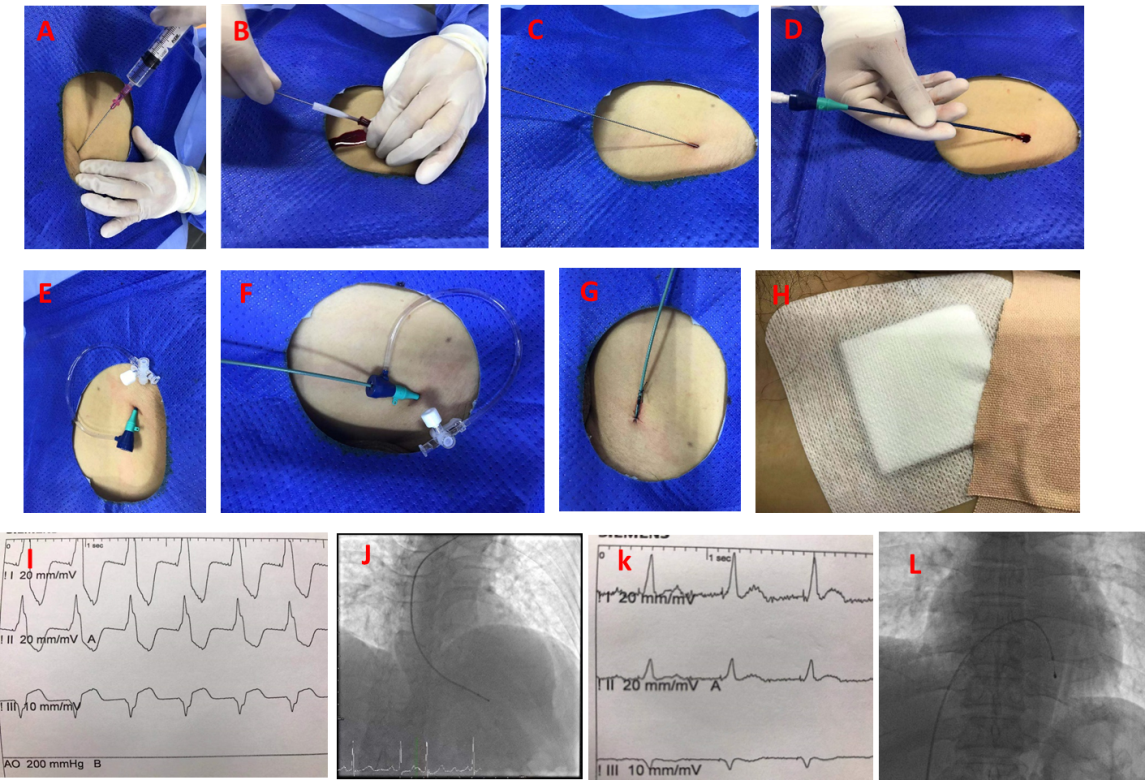
**

1. disinfected and locally anesthetized.

(B-C) A guide wire was inserted after puncturing the femoral or subclavicular vein

(D-E) A 6F sheath tube was inserted along the guide wire

(F) A temporary PM electrode was delivered to the right ventricular apex through the vein system

(G-K) Fixed the electrode head position and connected an external PM (G, H, I, and J for the S-control group and H, K, and L for the F-control group).

**Supplementary Table 1. Comparison of demographic and basal characteristics of patients between groups**

|  | F-control  n=107 | S-control  n=67 | N-group  n=105 | P value |
| --- | --- | --- | --- | --- |
| Age | 67.7±7.4 | 69.6±6.3 | 67.4±7.5 | 0.120 |
| Male | 67(62.6) | 43(64.2) | 63(60) | 0.848 |
| Sinus bradycardia | 66(61.7) | 43(64.2) | 66(62.9) | 0.946 |
| Sinus arrest  II°II AVB | 20(18.7)  15(14.0) | 12(17.9)  9(13.4) | 20(19.0)  14(13.3) | 0.983  0.988 |
| III°AVB | 6 (5.6) | 3(4.4) | 5(4.8) | 0.935 |
| Creatinine (μmol/L) | 79.0±14.0 | 80.5±14.0 | 78.8±16.2 | 0.724 |
| BUN (mmol/L) | 6.3±1.6 | 6.5±1.6 | 6.3±1.4 | 0.828 |
| Myoglobin (ng/mL) | 48.5±11.4 | 45.3±10.5 | 48.6±11.23 | 0.112 |
| Troponin (ng/mL) | 0.011±0.007 | 0.012±0.008 | 0.098±0.007 | 0.117 |
| CK-MB (ng/mL)  LDH (U/L) | 2.32±1.3  174.0±33.6 | 2.00±1.2  173.0±34.5 | 2.10±1.3  171.7±30.9 | 0.250  0.878 |
| WBC (10^9^/L) | 6.2±1.6 | 6.1±1.7 | 6.2±1.5 | 0.743 |
| HGB (g/L) | 148.7±10.4 | 148.2±11.6 | 150.1±11.0 | 0.209 |
| PLT (10^9^/L) | 225.5±59.0 | 234.3±63.3 | 216.6±61.0 | 0.172 |
| BNP (pg/mL) | 45.0±26.3 | 43.0±24.3 | 49.1±27.2 | 0.293 |
| [Tricuspid regurgitation](javascript:;) | 0 | 0 | 0 |  |

Data are mean ± SD. Data analysis was performed using the independent samples t-test. Abbreviation: BUN, blood urea nitrogen; CK-MB: Creatine kinase-MB; LDH: Lactate dehydrogenase; WBC, white blood cells; HGB, hemoglobin; PLT: platelets

**Supplementary Table 2. Comparison of laboratory test results between groups after temporary PM implantation**

|  | F-control  n=107 | S-control  n=67 | N-group  n=105 | P value |
| --- | --- | --- | --- | --- |
| Creatinine (μmol/L) | 78.1±10.9 | 76.3±11.5 | 80.0±12.0 | 0.114 |
| BUN (mmol/L) | 6.1±1.5 | 6.1±1.6 | 6.2±1.5 | 0.957 |
| Myoglobin (ng/mL) | 45.4±11.1 | 46.8±11.9 | 45.8±11.6 | 0.731 |
| Troponin (ng/mL) | 0.011±0.5 | 0.012±0.008 | 0.010±0.006 | 0.534 |
| CK-MB (ng/mL)  LDH (U/L) | 2.08±1.28  174.7±32.9 | 2.14±1.41  172.9±32.8 | 2.18±1.30  175.1±34.1 | 0.846  0.907 |
| WBC (10^9^/L) | 6.2±1.7 | 5.9±1.6 | 6.2±1.6 | 0.498 |
| HGB (g/L) | 149.7±10.9 | 149.8±10.9 | 150.1±10.5 | 0.961 |
| PLT (10^9^/L) | 223.3±59.2 | 229.2±64.7 | 217.7±62.1 | 0486 |
| BNP (pg/mL) | 42.7±23.3 | 48.6±27.1 | 50.8±26.3 | 0.073 |

Data are mean ± SD. Data analysis was performed using the independent samples t-test. Abbreviation: BUN, blood urea nitrogen; CK-MB: Creatine kinase-MB; LDH: Lactate dehydrogenase; WBC, white blood cells; HGB, hemoglobin; PLT: platelets

**Supplementary Videos**

**From the left median cubital vein**

**From the right median cubital vein**
